# BONCAT-iTRAQ Labelling Reveals Molecular Markers of Adaptive Responses in *Toxoplasma gondii* to Pyrimethamine Treatment

**DOI:** 10.3390/pathogens13100879

**Published:** 2024-10-08

**Authors:** John G. Mina, Anutthaman Parthasarathy, Exequiel O. Porta, Paul W. Denny, Karunakaran Kalesh

**Affiliations:** 1 Syngenta International Research Centre, Jealott’s Hall, Bracknell, Berkshire RG42 6EY, UK; john.mina@syngenta.com; 2 School of Chemistry and Life Sciences, Richmond Building, University of Bradford, Bradford BD7 1DP, UK; a.parthasarathy@bradford.ac.uk; 3 School of Pharmacy, University College London, London WC1N 1AX, UK; e.porta@ucl.ac.uk; 4 Department of Biosciences, Durham University, Durham DH1 3LE, UK; p.w.denny@durham.ac.uk; 5 School of Health and Life Sciences, Teesside University, Middlesbrough TS1 3BX, UK; 6 National Horizons Centre, 38 John Dixon Lane, Darlington DL1 1HG, UK

**Keywords:** *Toxoplasma gondii*, BONCAT, iTRAQ, quantitative proteomics, pyrimethamine

## Abstract

We employed a BONCAT-iTRAQ labelling approach to investigate newly synthesised proteins (NSPs) in *Toxoplasma gondii* subjected to varying concentrations of the antifolate drug pyrimethamine. Our results reveal that numerous NSPs exhibited altered expression levels in response to the drug, with significant upregulation observed at higher concentrations. Key proteins involved in protein synthesis, stress responses, energy metabolism, and cytoskeletal dynamics were identified, indicating that *T. gondii* undergoes complex adaptive responses to pyrimethamine treatment. While some of the identified pathways reflect a generic stress response, this study provides important molecular markers and mechanistic insights specific to the parasite’s adaptation strategies. These findings contribute to understanding how *T. gondii* modulates its proteome in response to drug-induced stress and lay the groundwork for further investigations into potential therapeutic targets.

## 1. Introduction

*Toxoplasma gondii* (*T. gondii*) is an obligate intracellular protozoan parasite responsible for toxoplasmosis, a widespread infection affecting nearly one-third of the world’s population [1]. While many infections are asymptomatic, toxoplasmosis can cause severe complications in immunocompromised individuals and pregnant women, leading to encephalitis, congenital defects, and even death in extreme cases. As such, effective treatment strategies are essential to control the disease.

**Figure 1:**
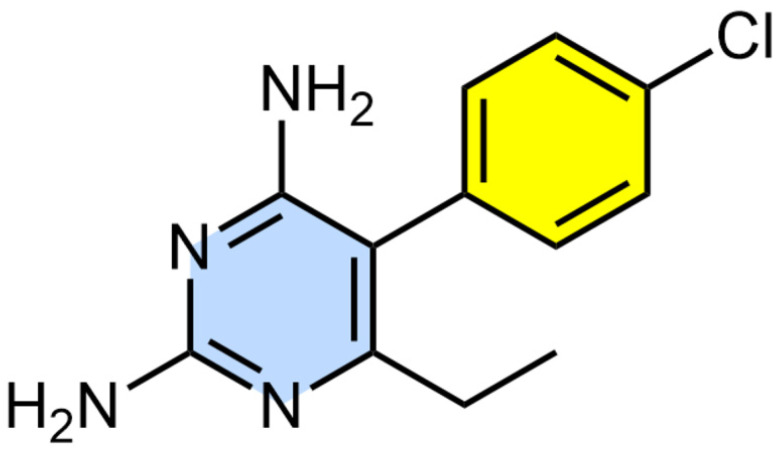
Chemical structure of pyrimethamine (PM).

One of the primary treatments for toxoplasmosis involves the use of pyrimethamine (PM) (Figure 1), an antiparasitic drug that targets dihydrofolate reductase (DHFR) within the folate metabolism pathway [2]. By inhibiting DHFR, PM disrupts the synthesis of tetrahydrofolate, an essential cofactor in the synthesis of nucleotides, thereby hindering DNA replication and cell division in the parasite. *T. gondii* demonstrates an ability to respond to drug pressure by activating transient adaptive mechanisms that may allow it to survive under treatment conditions. While resistance to PM and other drugs has been well-documented in the literature, primarily through genetic mutations and selection pressures [3,4,5,6], our study suggests that non-mutational, compensatory responses such as changes in protein expression also play a role in the parasite’s survival during drug exposure. Understanding these adaptive responses is crucial for improving therapeutic strategies and combating drug resistance.

Compensatory mechanisms often involve the upregulation or activation of alternative metabolic pathways, stress response proteins, and invasion-related proteins. These processes enable the parasite to maintain its essential functions despite the disruption of primary metabolic pathways. Investigating these compensatory responses can reveal potential targets for combination therapies that may enhance the efficacy of existing treatments and prevent the development of resistance.

**Figure 2:**
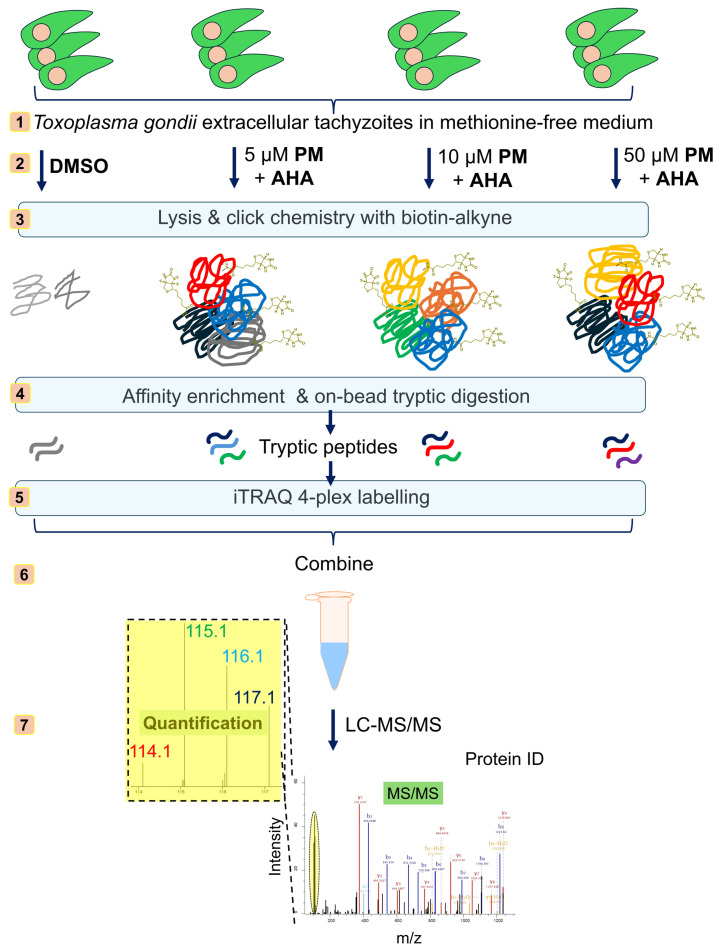
Schematic overview of PM treatment combined with Bioorthogonal Non-Canonical Amino Acid Tagging (BONCAT) metabolic labelling and Isobaric Tags for Relative and Absolute Quantification (iTRAQ)-based quantitative proteomics mass spectrometry for the profiling of the effect of PM treatment on newly synthesised proteins (NSPs) in *Toxoplasma gondii*.

Traditional proteomics approaches provide a snapshot of the entire proteome but often overlook dynamic changes in newly synthesised proteins (NSPs) that are critical for the parasite’s adaptive responses. To address this limitation, we employed Bioorthogonal Non-Canonical Amino Acid Tagging (BONCAT) [7,8] in combination with Isobaric Tags for Relative and Absolute Quantification (iTRAQ) labelling [9,10]-based quantitative proteomics (Figure 2), as we previously reported in *Leishmania* spp. parasites [11,12]. BONCAT allows for the selective tagging and enrichment of NSPs, providing insights into the proteins that are actively synthesised in response to drug treatment. iTRAQ labelling enables the multiplexed quantification of these proteins, allowing for a robust relative comparison of their expression levels under different treatment conditions.

In this study, we aimed to identify and quantify the NSPs in *T. gondii* treated with varying concentrations of PM, revealing the concentration-dependent effects of the drug on the parasite’s proteome. Our findings highlight the key proteins and pathways involved in *T. gondii*’s adaptive response to PM, offering potential targets for therapeutic intervention.

## 2. Materials and Methods

### 2.1. T. gondii Culture

*T. gondii* tachyzoites were maintained by passage in human foreskin fibroblast (HFF; SRC-1041 from ATCC, Manassas, VA, USA) cells in complete medium (Dulbecco’s Modified Eagle Medium (DMEM) supplemented with 10% heat-inactivated foetal bovine serum and 2 mM L-glutamine) as previously described [13]. Briefly, the parasites were allowed to grow until they lysed the host cells, indicating the successful completion of the replication cycle. Upon lysis of the host cells, the cell debris and parasite mixture from eight T-25 flasks (Fisher Scientific, Cambridge, UK) were combined to ensure sufficient biomass for subsequent experiments. The mixture was homogenised by passing it through a syringe equipped with a G25 needle (Fisher Scientific, Cambridge, UK) six times. The mixture was then filtered through a 3 µm filter to remove host cell debris while retaining the free tachyzoites. Following filtration, the parasite suspension was quantified, yielding a total volume of 40 mL with a concentration of 8 × 10^6^ parasites per mL. The parasite suspension was then centrifuged at 800× *g* for 10 min at 4 °C to pellet the tachyzoites. The supernatant was carefully removed, and the parasite pellet was resuspended in 12 mL of fresh methionine-free DMEM supplemented with 10% heat-inactivated foetal bovine serum and L-glutamine. The resuspended parasites were divided into twelve equal samples, each containing 1 mL of the suspension, corresponding to approximately 2.7 × 10^7^ *T. gondii* cells per sample.

### 2.2. BONCAT Labelling and PM Treatment

Each of the twelve parasite samples was incubated under standard culture conditions (37 °C, 5% CO_2_, and 95% relative humidity) for 45 min to allow the parasites to acclimate in the methionine-free medium. Following incubation, the samples were concurrently treated in triplicate with 200 µM azidohomoalanine (AHA) and with varying concentrations of PM (TOCRIS; 50 µM, 10 µM, 5 µM, and DMSO control) for 1 h. The treatment of AHA at a 200 µM final concentration was based on optimal labelling results in our earlier BONCAT experiments [11,12]. For the PM treatments, the concentrations used are consistent with ranges commonly used in in vitro studies assessing the efficacy of the drug in parasite treatment, as these doses are known to exhibit varying levels of parasite inhibition while minimising toxicity to the host [14]. After the treatment, the samples were centrifuged at 1600× *g* for 5 min at room temperature to pellet the tachyzoites. The supernatant was carefully removed, and the parasite pellets were washed twice with phosphate buffered saline (PBS) and then lysed using 200 µL of lysis buffer (50 mM 4-(2-hydroxyethyl)piperazine-1-ethanesulfonic acid (HEPES) at pH 7.4, 150 mM NaCl, 4% sodium dodecyl sulphate (SDS), and 250 U benzonase).

### 2.3. Click Chemistry-Based Proteome Enrichment, On-Bead Processing and iTRAQ Labelling for Quantitative Analysis

The *T. gondii* lysates were subjected to click reactions with Biotin-Alkyne (Acetylene-PEG4-Biotin; Jena Bioscience, Löbstedter Str. 71, Jena, Germany) using our optimised click reaction conditions as reported earlier [11,12]. Briefly, the lysates were treated for 3 h at room temperature with a freshly prepared click chemistry reaction cocktail containing the following components: 100 µM Biotin-Alkyne in DMSO, 1 mM copper sulphate solution in water, 1 mM Tris (2-carboxyethyl)phosphine hydrochloride (TCEP) solution in water, and 100 µM Tris [(1-benzyl-1H-1,2,3-triazol-4-yl)methyl]amine (TBTA) solution in DMSO. The click reactions were then stopped by adding 10 mM ethylenediaminetetraacetic acid (EDTA) solution and the proteins were precipitated by adding 4 volumes of methanol, 1.5 volumes of chloroform, and 3 volumes of water to the reaction mixtures. The solutions were vortexed thoroughly for 1 min and centrifuged at 16,000× *g* for 5 min at room temperature, and the protein pellets were collected. The protein pellets were washed twice with 10 volumes of ice-cold methanol and the samples were air-dried for 10 min.

The air-dried protein pellets were resuspended in PBS containing 2% SDS via sonication to a concentration of 5 mg/mL. A typical experiment involved 300 µg of *T. gondii* lysate, which was diluted 20-fold with PBS to reduce the SDS content to 0.1%. Insoluble debris was removed by centrifugation at 10,000× *g* for 5 min. NeutrAvidin-Agarose beads (30 µL; Fisher Scientific, Cambridge, UK), pre-washed three times with 0.1% SDS in PBS, were added to each sample. These mixtures were rotated for 1.5 h at room temperature. The beads were washed three times with 1% SDS in PBS, once with 6 M urea, three times with PBS, and once with 25 mM triethylammonium bicarbonate (TEAB), with centrifugation at 2000× *g* for 1 min between washes. The beads were then resuspended in 150 µL of 25 mM TEAB and treated with 5 mM TCEP at 50 °C for 30 min. After a quick wash with 25 mM TEAB, the beads were resuspended in 150 µL of 25 mM TEAB and alkylated with 10 mM chloroacetamide (CAA) in the dark at room temperature for 30 min. The beads were again washed and subjected to an overnight digestion with 5 µg of sequencing-grade modified trypsin at 37 °C in 200 µL of 50 mM TEAB. The digested peptides were collected by centrifugation at 5000× *g* for 5 min. The beads were washed with 100 µL of 50% acetonitrile (ACN) and 0.1% formic acid (FA), and the wash was pooled with the digested peptides. The pooled peptide samples were acidified to pH 3 using FA and dried under vacuum. The peptides were then redissolved in 0.1% (*v*/*v*) FA solution in water and subjected to desalting on Pierce™ C-18 Spin Columns (Fisher Scientific, Cambridge, UK; CN: 89873) following the manufacturer’s instructions. The peptides were then evaporated to complete dryness under vacuum.

The tryptic peptides were then labelled with iTRAQ reagents using the iTRAQ Reagents Multiplex Kit (Sigma-Aldrich, Dorset, UK), following the manufacturer’s protocol, as described earlier [11,12]. Briefly, each tryptic peptide sample was resuspended in 30 µL of dissolution buffer (0.5 M TEAB buffer supplied with the iTRAQ Kit). A total of 210 µL of absolute ethanol was added to each iTRAQ 4-plex reagent vial equilibrated to room temperature. A total of 70 µL of the contents from each iTRAQ reagents vial was quickly added to the respective vials of peptide digests. The step was repeated for the other two replicates. The labelling reactions were performed for 1.5 h at room temperature, following which the reactions were quenched with 100 mM Tris base for 15 min at room temperature. The samples labelled with the four different iTRAQ channels were then pooled into a fresh vial and concentrated under vacuum. The peptides were reconstituted in water with 0.1% (*v*/*v*) FA and 2% (*v*/*v*) ACN and subjected to desalting on C-18 Sep-Pak Classic cartridges (Waters Ltd., Wilmslow, UK; WAT051910) following the manufacturer’s instructions. The eluted peptides were concentrated to dryness under vacuum and subjected to a second round of cleaning on HILIC TopTip (PolyLC Inc., Columbia, MD, USA; TT200HIL) solid-phase extraction tips following the manufacturer’s instructions. The eluted peptides were concentrated under vacuum and reconstituted in aqueous 0.1% (*v*/*v*) FA.

### 2.4. Liquid Chromatrography-Tandem Mass Spectrometry (LC-MS/MS) Analysis

Labelled peptides were separated using an ekspert^TM^ nanoLC 425 system (Eksigent Technologies LLC, Dublin, CA, USA) equipped with a YMC-Triart C18 column (150 × 0.3 mm) preceded by a C-18 trap column. A gradient of LC-MS grade water with 0.1% FA (mobile phase A) and ACN with 0.1% FA (mobile phase B) was used over an 87 min period at a flow rate of 5 µL/min. The separated peptides were analysed on a Sciex TripleTOF 6600 system (Alderley Park, Macclesfield, UK) in high-resolution mode. MS1 spectra were acquired over a mass range of 400–1600 *m*/*z* and MS/MS spectra were collected from 100 to 1500 *m*/*z* for up to 30 precursors per cycle.

### 2.5. Data Analysis

The MS data were processed using MaxQuant software (version 1.6.3.4) [15] with the integrated Andromeda search engine [16]. *T. gondii* protein sequences were retrieved from the UniProt KB database and searched with the following parameters: trypsin digestion, iTRAQ 4-plex labelling, carbamidomethylation of cysteine (fixed modification), oxidation of methionine and N-terminal acetylation (variable modifications), with a maximum of 2 missed cleavages and 5 modifications per peptide. FDR thresholds were set at 0.01. Further data analysis, including normalisation, statistical analysis, and the generation of volcano plots, was carried out using Perseus software (version 1.6.2.3) [17]. Volcano plots were generated in R 64-bit version 4.2.3 using the R packages ggplot2 (version 3.4.2) and EnhancedVolcano (version 1.16.0). Protein–protein interaction network analyses were performed by using the publicly available STRING database [18] of *T. gondii*. Cytoscape (version 3.10.2) [19] was used for refining, analysing, and visualising the protein interaction networks.

## 3. Results

### 3.1. BONCAT-iTRAQ Profiling of PM-Induced Changes in Global Nascent Protein Synthesis in T. gondii

**Figure 3:**
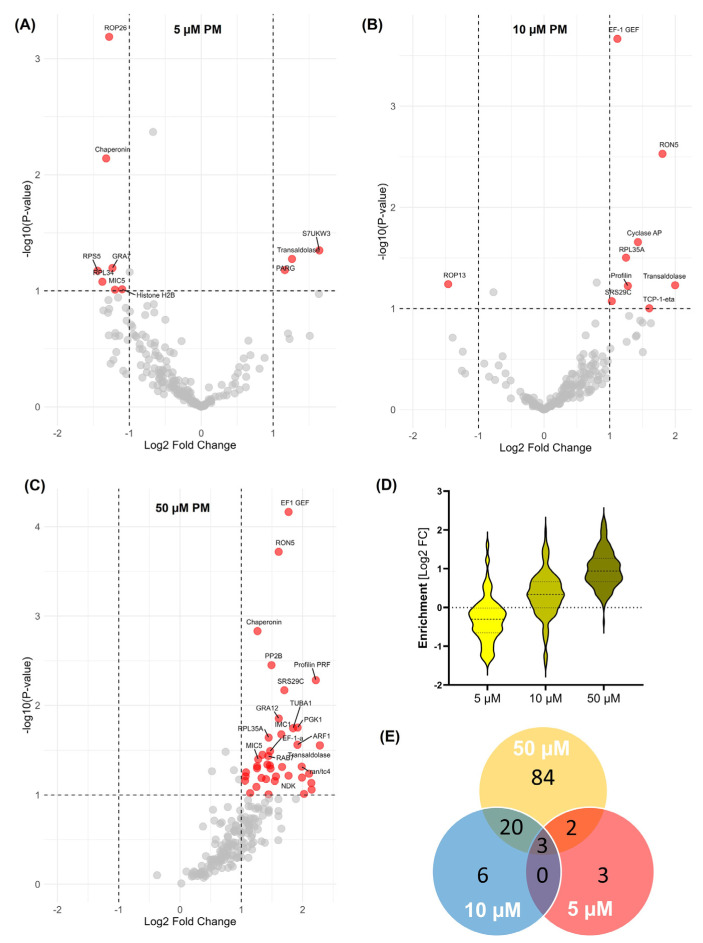
Characterisation of the effect of pyrimethamine treatment (PM) on nascent protein synthesis in *Toxoplasma gondii*. (**A**) Volcano plots show significant enrichment of newly synthesised proteins at 5 µM (**B**), 10 µM (**C**), and 50 µM (**D**) PM treatment over that with vehicle (DMSO) treatment across three replicates. A modified *t* test with permutation-based FDR statistics was applied (250 permutations, FDR = 0.05) to compare PM-treated and vehicle-treated groups. (**D**) Violin plot demonstrating the PM concentration-dependent dynamic changes in global nascent protein synthesis. (**E**) Venn diagram showing the number of nascent proteins enriched at 5 µM (red), 10 µM (blue), and 50 µM (orange) PM treatments.

The BONCAT-iTRAQ-based quantitative proteomics revealed significant alterations in the expression of 218 NSPs across three replicates in *T. gondii* in response to PM treatment (Table S1). We observed differential expressions of NSPs in response to varying concentrations of PM (Figure 3), suggesting a complex, dose-dependent response mechanism in *T. gondii*. Interestingly, decreases in the expression of several NSPs were noted at a low dose (5 µM) of PM treatment (Figure 3A). This suggests that at a low PM dose, *T. gondii* enters a state of metabolic conservation, downregulating processes related to translation, invasion, and stress response. This could be a survival strategy to minimise the impact of drug exposure. The top hits in the downregulated proteins (Table 1) include several ribosomal proteins, rhoptry and microneme proteins, chaperonins, and proteins involved in cell division and protein folding. Interestingly, a small subset of NSPs including poly(ADP-ribose) glycohydrolase (PARG), S15 sporozoite-expressed protein, alveolin domain-containing intermediate filament IMC10, transaldolase, IMP2_N domain-containing protein, and acid phosphatase GAP50 showed increased expression at the low dose of PM treatment (Table 1).

We observed a clear trend of a PM-concentration-dependent increased expression of several NSPs in *T. gondii* (Figure 3B–D). The Venn diagram (Figure 3E) illustrates the overlap and distinct sets of nascent proteins enriched under different concentrations of PM treatment as well as the proteins shared across the treatment conditions. The top upregulated NSPs at a high dose of PM include transaldolase, profilin, S15 sporozoite-expressed protein, rhoptry neck protein RON5, chaperonin S7UXE3, serine/threonine-protein phosphatase PP2B, EF-1 guanine nucleotide exchange domain-containing protein (EF1 GEF), phosphoglycerate kinase (PGK), adp ribosylation factor ARF1, elongation factor 1-alpha (EF-1α), and RAB7 GTPase and GTP-binding nuclear protein Ran/Tc4 (Table 1). This dynamic regulation underscores the parasite’s ability to modulate its biological processes in a dose-dependent manner, highlighting the complexity of its adaptive strategies in response to drug treatment.

### 3.2. Protein–Protein Interaction Network Analysis of PM-Modulated NSPs in T. gondii

**Figure 4:**
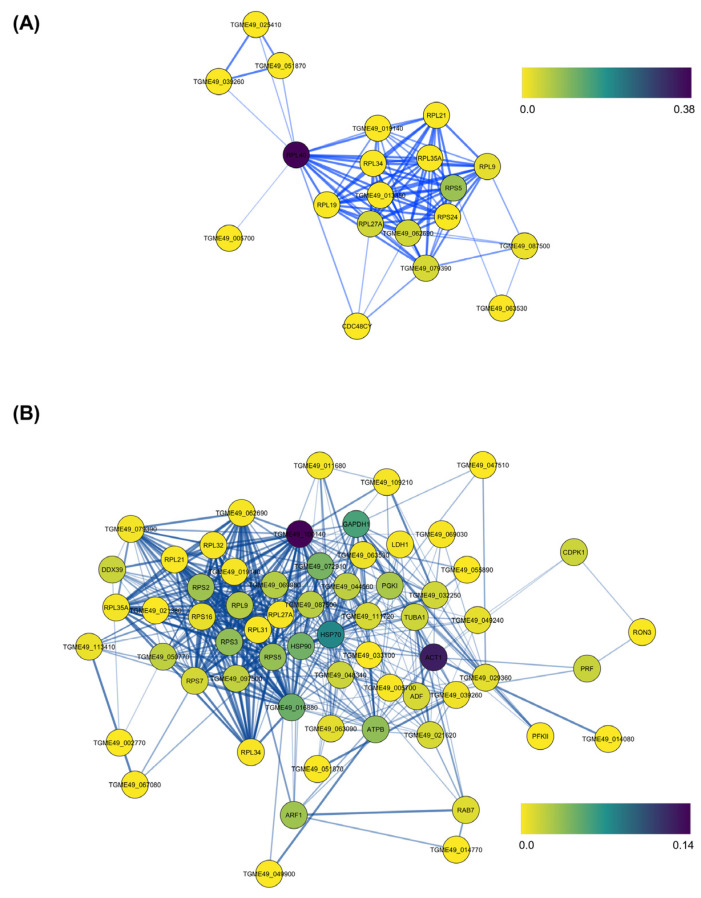
Protein–protein interaction network of newly synthesised proteins (NSPs) in *Toxoplasma gondii* (**A**) downregulated upon treatment of 5 µM pyrimethamine (PM) and (**B**) upregulated upon treatment of 50 µM PM constructed using the publicly available STRING database. The nodes are coloured according to their betweenness centrality in the network.

An analysis of protein–protein interaction (PPI) networks of the PM-perturbed *T. gondii* NSPs revealed significant nodes in terms of betweenness centrality, which identifies the most influential proteins in network communication (Table S2). In the network of downregulated proteins upon low-dose PM treatment (Figure 4A), ribosomal-ubiquitin protein RPL40 and ribosomal proteins RPS5 and RPL27A showed the highest betweenness centrality. This suggests that under low-dose PM, *T. gondii* reduces its overall metabolic activity, possibly for entering a more dormant state to conserve energy and reduce unnecessary protein turnover. The network of upregulated proteins upon high-dose PM treatment (Table S3) showed the highest betweenness centrality for elongation factor 1-gamma (EF1-γ), actin, heat shock protein HSP70, glyceraldehyde-3-phosphate dehydrogenase (GAPDH1), and guanine nucleotide-binding protein (G-Protein) (Figure 4B). This suggests that *T. gondii* upregulates pathways related to protein synthesis, cytoskeletal integrity, protein folding and stress responses, glycolysis, and cellular signalling to counteract the stress from PM.

## 4. Discussion

This study reveals *T. gondii*’s ability to modulate its cellular processes depending on the severity of PM challenge. The top downregulated ribosomal proteins (RPS5, RPL34, RPL21, RPL27, RPL9, RPL27A, and RPS15) observed at low PM doses are essential for protein synthesis [20]. The downregulation of these proteins suggests a suppression of global protein synthesis, likely as an energy-saving response to moderate metabolic stress. Similarly, the downregulated chaperonins (putative chaperonin and CCT-theta) assist in the proper folding of NSPs, ensuring their three-dimensional structure and functionality. Their initial downregulation suggests a decreased need for protein folding due to reduced protein synthesis under low-dose stress.

The downregulated rhoptry and microneme proteins (ROP13, ROP26, ROP4, MIC5) are key players in the invasion process of host cells by *T. gondii* [21]. These proteins are involved in the formation of the parasitophorous vacuole and the subsequent invasion and survival within the host. Their decreased expression at low PM doses suggests a strategic downregulation of the invasion machinery for conserving energy under sub-lethal drug stress. The downregulated cell division and stress response proteins (CDC48CY, ubiquitin, and cyclophilin) are involved in cell division and the ubiquitin–proteasome system, which is critical for protein degradation and quality control [22]. Ubiquitin tags proteins for degradation, while cyclophilins are involved in protein folding and immune modulation. The decrease at low drug doses suggests a temporary halt in cell division and protein degradation processes. Histone H2B is a component of nucleosomes, playing a crucial role in DNA packaging and gene regulation. Seryl-tRNA synthetase is involved in charging tRNAs with serine, a critical step in translation [23]. The coordinated regulation of these proteins indicates changes in gene expression and translation rates in response to PM treatment.

The increased expression of a specific set of NSPs at a low dose of PM treatment suggests a nuanced adaptive response by *T. gondii*. PARG is involved in the regulation of poly(ADP-ribosyl)ation: a post-translational modification critical in DNA repair and cellular stress responses [24,25]. The increased expression of PARG at low PM doses suggests that the parasite might be experiencing low-level DNA damage or stress, prompting the activation of repair mechanisms. This might be a protective measure to maintain genomic integrity and ensure survival under sub-lethal drug stress. IMC10 is a component of the inner membrane complex (IMC), which plays a role in maintaining the structural integrity of the parasite and its motility, especially during invasion and replication [26]. The upregulation of IMC10 at low drug concentrations indicates a need to reinforce the parasite’s cytoskeletal framework, potentially as a preparatory measure to cope with the early stress induced by the drug. Transaldolase is involved in the pentose phosphate pathway, which is crucial for nucleotide synthesis and redox balance [27]. The upregulation of transaldolase suggests an attempt by the parasite to modulate its metabolic pathways to compensate for the metabolic stress induced by PM. This could be part of a broader strategy to ensure the adequate production of nucleotides and maintain the redox state within the cell. The upregulation of S15 sporozoite-expressed protein reflects an early-stage stress response or a shift in the parasite’s developmental programme [28]. While PM targets the tachyzoite stage, the expression of S15 sporozoite-associated protein suggests an attempt by the parasite to enter a dormant or alternative stage as a survival strategy under drug pressure.

The upregulation of both EF1 GEF and EF-1α upon higher doses of PM treatment suggests an important adaptive response by *T. gondii*. These proteins are critical components of the protein synthesis machinery, and their increased expression can be linked to several adaptive and compensatory mechanisms in response to the stress induced by PM. EF-1α plays a crucial role in the elongation phase of protein synthesis by facilitating the binding of aminoacyl-tRNA to the ribosome [29]. Increased levels of EF-1α suggest that the parasite is attempting to boost its protein synthesis capacity, possibly to counteract the impaired cellular functions caused by PM. This would allow the parasite to produce essential proteins required for survival and adaptation under drug-induced stress. Similarly, EF1 GEF is responsible for the regeneration of active EF-1α by catalysing the exchange of GDP for GTP [30]. The upregulation of EF1 GEF ensures a continuous supply of active EF-1α, further supporting the increased demand for protein synthesis. It is likely that high doses of PM induce ribosomal stress, necessitating the upregulation of factors involved in maintaining ribosome function and protein translation. EF-1α and EF1 GEF are key players in the translation process, and their upregulation helps maintain ribosomal activity and protein synthesis efficiency under conditions of drug-induced stress.

Profilin is an actin-binding protein critical for the regulation of the actin cytoskeleton and host cell invasion. In *T. gondii*, profilin also serves as an immunodominant antigen, eliciting strong immune responses from the host [31]. The enrichment of profilin indicates that PM treatment may influence the parasite’s invasion mechanisms, potentially as a compensatory response to the drug’s inhibitory effects. The enhanced expression of profilin could help the parasite maintain its motility and invasion capabilities, ensuring its survival under drug pressure. Additionally, profilin’s role in immune modulation suggests a possible strategy by the parasite to evade the host immune response or manage drug-induced stress [32].

The upregulated rhoptry neck protein RON5 is part of the rhoptry neck complex, which is essential for the parasite’s attachment to and invasion of host cells [33]. The upregulation of this protein suggests that *T. gondii* is ramping up invasion-related processes in response to high-dose PM treatment. The upregulated chaperonin (S7UXE3) assists in the proper folding of proteins under stress conditions, preventing misfolding and aggregation. The upregulation of this protein suggests that PM treatment is inducing a stress response, prompting *T. gondii* to enhance its protein quality control mechanisms. By increasing the synthesis of chaperonins, the parasite may be managing the accumulation of misfolded or damaged proteins resulting from disrupted cellular processes.

The upregulated serine/threonine-protein phosphatase 2B (PP2B), a protein that belongs to the calcineurin family, is a calcium/calmodulin-dependent protein phosphatase that plays a critical role in various cellular processes, including stress response, signal transduction, and the regulation of gene expression [34]. It is likely that the cellular stress induced by PM leads to alterations in intracellular calcium levels. This could activate calcineurin, which then dephosphorylates target proteins involved in stress responses, allowing the parasite to adapt to the drug-induced stress. Calcineurin is also involved in processes such as vesicular trafficking and invasion, which are critical for the parasite’s life cycle [35]. By modulating the phosphorylation state of proteins, *T. gondii* may be augmenting these pathways to ensure the successful invasion of host cells and survival within the host environment, even under the stress of PM treatment.

PGK is a key enzyme in the glycolytic pathway, catalysing the conversion of 1,3-bisphosphoglycerate to 3-phosphoglycerate, generating ATP in the process [36]. The upregulation of PGK indicates an increased demand for ATP production, which is crucial for maintaining energy homeostasis, especially under the stress induced by PM. Since PM disrupts nucleotide synthesis through the inhibition of DHFR, *T. gondii* may compensate by upregulating glycolysis to ensure a sufficient energy supply for other cellular processes, including stress responses and repair mechanisms.

ARF1 is involved in regulating vesicular trafficking, including the formation of coated vesicles and the transport of proteins between the Golgi apparatus and other organelles [37,38]. The upregulation of ARF1 suggests that *T. gondii* might be enhancing its vesicular transport mechanisms to ensure the proper distribution of proteins, lipids, and other molecules, which is essential for maintaining cellular functions under stress. ARF1 may also play a role in the trafficking of membrane proteins involved in drug resistance or stress responses. Enhancing vesicular trafficking may help the parasite efficiently manage and deliver proteins required for counteracting PM’s effects.

Ran/TC4 is involved in the regulation of nuclear-cytoplasmic transport, including the import and export of proteins and RNA across the nuclear envelope [39]. The upregulation of Ran/TC4 indicates an increased need for efficient nuclear transport, which may be vital for regulating gene expression and responding to stress at the transcriptional level. Ran is also known to play roles in cell cycle progression and mitotic spindle assembly [40]. Its upregulation could be linked to *T. gondii*’s efforts to maintain cell cycle progression and proper mitotic function under the stress of PM, ensuring the parasite’s continued survival and proliferation.

The RPL40 observed with the highest betweenness centrality at low PM doses in the protein–protein interaction network is a fusion protein that combines ubiquitin with ribosomal protein L40 [41]. The centrality of RPL40 suggests that degradation pathways and protein synthesis regulation are downregulated under low-dose PM. This indicates a reduced priority for protein turnover or repair at this concentration. The EF1-γ observed with the highest betweenness centrality at high PM doses in the protein–protein interaction network is involved in protein synthesis by acting as part of the elongation factor complex that facilitates the binding of aminoacyl-tRNAs to the ribosome [42]. Its high centrality suggests that translation elongation and protein synthesis become critical processes during *T. gondii*’s adaptation to high-dose PM treatment. This aligns with the need for increased protein turnover and the synthesis of stress response proteins. Similarly, GAPDH is a key enzyme in glycolysis [43], and its role in maintaining the energy metabolism is crucial under PM stress. The centrality of GAPDH1 suggests that energy metabolism, particularly glycolysis, is heavily involved in the parasite’s adaptive response to maintain ATP levels and cellular homeostasis. Another important protein with high betweenness centrality in the network, the G-protein, is involved in signal transduction pathways and plays crucial roles in various cellular processes, including cell proliferation, differentiation, and survival [44]. Their high centrality implies that signalling pathways are being heavily utilised for adaptive responses and survival strategies in the face of high-dose PM.

It is well-established that *T. gondii*, as an obligate intracellular parasite, primarily relies on the intracellular environment of the host cell to activate its full protein synthesis machinery. However, studies have shown that *T. gondii* can remain metabolically active and continue protein synthesis in the extracellular environment for short periods before it invades a new host cell [45,46]. In this study, we used extracellular parasites to perform BONCAT-iTRAQ labelling, which allowed us to probe the translation machinery and assess NSPs in response to PM treatment. While we acknowledge that this approach may overlook some candidate proteins that are preferentially or exclusively synthesised in the intracellular environment, our findings demonstrate that key components of the translation machinery, stress response pathways, and adaptive mechanisms remain highly active even in the absence of host cell interaction. This is particularly relevant to understanding how *T. gondii* prepares for host cell invasion or adapts to drug-induced stress, as these processes may be initiated extracellularly before full intracellular activity resumes. Additionally, our results provide a snapshot of protein synthesis in *T. gondii* during the extracellular stage, revealing insights into how the parasite responds to drug pressure when outside the host cell. These findings pave the way for future studies where the BONCAT-quantitative proteomics combination will be applied to intracellular parasites to capture a more comprehensive profile of NSPs synthesised under intracellular conditions. This will complement and expand upon the results presented here.

Thus, the findings of this BONCAT-iTRAQ labelling study reveal a complex adaptive response by *T. gondii* to PM treatment. The concentration-dependent enrichment of proteins involved in invasion, stress response, protein synthesis, and metabolic adaptation highlights the parasite’s ability to dynamically regulate its proteome in response to drug-induced challenges. The upregulation of invasion-related proteins such as profilin and RON5 suggests that *T. gondii* may be prioritising mechanisms that ensure its continued ability to invade host cells, a critical step for its survival and propagation. The enrichment of translation-related proteins, including EF-1 GEF and EF-1α, indicates an effort to maintain protein synthesis under drug pressure, potentially to sustain the production of essential proteins despite the disruption of folate metabolism. The increased expression of stress-response proteins, such as chaperonins and PP2B, underscores the parasite’s capacity to manage the cellular stress induced by PM. The upregulation of transaldolase, a key enzyme in the PPP, suggests a shift towards metabolic pathways that support nucleotide synthesis and redox balance, which is crucial for the parasite’s survival under drug treatment.

## 5. Conclusions

This study provides valuable insights into the adaptive mechanisms employed by *T. gondii* in response to PM treatment. The concentration-dependent increase in certain NSPs suggests that *T. gondii* actively modulates its proteome to counteract the effects of PM, employing strategies that enhance its invasion capacity, stress tolerance, and metabolic flexibility. The identification of key proteins involved in these adaptive responses offers potential targets for future therapeutic interventions. For example, targeting profilin and RON5 could disrupt the parasite’s ability to invade host cells, while inhibiting chaperonins and serine/threonine-protein phosphatases might enhance the efficacy of PM by exacerbating cellular stress. Additionally, targeting the pentose phosphate pathway, as suggested by the enrichment of transaldolase, could further impair the parasite’s ability to manage oxidative stress and maintain nucleotide synthesis.

This dynamic regulation underscores *T. gondii*’s ability to modulate its biological processes in a dose-dependent manner, highlighting the complexity of its adaptive strategies in response to drug treatment. However, the findings of this study require further validation through functional assays and in vivo studies to confirm the roles of the identified proteins in PM resistance and adaptive responses. Future studies should also explore the broader implications of these findings for understanding drug resistance mechanisms in *T. gondii* and other apicomplexan parasites.

Overall, the combination of BONCAT and iTRAQ labelling represents a powerful approach for studying dynamic proteomic changes in response to drug treatment, offering new avenues for understanding and combating drug resistance in parasitic infections.

## Figures and Tables

**Table 1:** Significantly enriched nascent proteins at 5 µM and 50 µM pyrimethamine treatment for 1 h in *Toxoplasma gondii* ^1^.

Protein Name	Protein ID	Log_2_ FC/5 µM	Log_2_ FC/50 µM
Transaldolase	S7V2K7	1.263	2.282
Profilin	S7UIQ6	0.656	2.216
S15 sporozoite-expressed protein	S7UKW3	1.643	2.147
Proteasome 26S regulatory subunit	S7W860	N/A	2.143
Lysine decarboxylase family protein	S7W5X6	N/A	2.105
IMC10	S7WBA5	1.229	2.024
Fructose-bisphosphatase	A0A125YGG2	N/A	1.991
GTP-binding nuclear protein	S7V0L0	N/A	1.986
Phosphoglycerate kinase	S7VZ21	N/A	1.920
ADP ribosylation factor ARF1	S7UMN8	N/A	1.916
T-complex protein 1 subunit eta	S7UKY6	N/A	1.891
Tubulin alpha chain	S7UL74	N/A	1.845
EF-1 GEF	S7UX25	−0.670	1.771
PyrDOX	S7VY56	N/A	1.771
Major surface antigen	E0AEY9	−0.499	1.702
Heat shock protein HSP70	A0A125YP12	N/A	1.665
IMC1	A0A125YFV6	N/A	1.653
Dense granule protein GRA12	S7VY87	N/A	1.612
Rhoptry neck protein RON5	S7W8Q7	−0.274	1.610
26S protease regulatory subunit 4	A0A125YSZ9	N/A	1.606
Preprotein translocase Sec61	A0A125YSW3	N/A	1.606
CDPK1	S7UHK8	N/A	1.606
Putative elongation factor 1-gamma	A0A125YG44	N/A	1.566
Nucleoside diphosphate kinase	A0A125YXT7	N/A	1.546
Serine/threonine-protein phosphatase	A0A125YWN7	−0.660	1.492
Putative transmembrane protein	S7ULN4	N/A	1.478
Elongation factor 1-alpha	S7UIZ8	−0.771	1.470
Rhoptry protein 5B	F2YGR7	−0.780	1.467
Protein disulphide-isomerase	A0A125YQI9	N/A	1.453
Putative heat shock protein 90	S7VTT7	N/A	1.451
Prolyl-tRNA synthetase	S7V2A6	N/A	1.448
Ribosomal protein RPL35A	A0A125YPQ6	−0.653	1.446
Rhoptry protein ROP15	S7UKJ6	N/A	1.444
GTPase RAB7	S7V0V9	N/A	1.441
Small GTP binding protein rab1a	S7W5S1	−0.643	1.425
DDX39	A0A125YNR1	N/A	1.404
GDPH	S7UQ54	N/A	1.386
Actin ACT1	A0A125YH17	N/A	1.360
Cyclophilin	A0A125YTE9	−0.995	1.345
Adenine nucleotide translocator	A0A125YLS5	N/A	1.330
RRM-containing protein	A0A125YHM1	N/A	1.284
Microneme protein MIC5	S7W847	−1.200	1.275
Putative chaperonin	S7UXE3	−1.322	1.264
Fructose-bisphosphate aldolase	A0A125YGE5	−0.903	1.258
ATP synthase subunit beta	A0A125YYY4	N/A	1.256
Rhoptry protein ROP7	A0A125YJ16	N/A	1.246
Rhoptry kinase family protein ROP26	S7UKG8	−1.281	1.077
Dense granule protein GRA7	A0A125YVB8	−1.235	1.071
Ribosomal protein RPL27A	A0A125YTM1	−1.050	1.061
Ribosomal protein RPS5	A0A125YNF8	−1.441	N/A
Ribosomal protein RPL34	A0A125YXF2	−1.375	N/A
Cell division protein CDC48CY	A0A125YX44	−1.361	N/A
Rhoptry protein ROP13	A0A125YQ57	−1.298	N/A
Ribosomal protein RPL21	S7WHD1	−1.291	N/A
Ribosomal protein RPL27	S7UYE2	−1.286	N/A
CCT-theta	S7UPG8	−1.280	N/A
Ribosomal protein RPS15	A0A125YHZ1	−1.195	N/A
Ubiquitin	A0A125YNY4	−1.195	N/A
Ribosomal protein RPL9	A0A125YRM1	−1.157	N/A
Seryl-tRNA synthetase	S7WG10	−1.139	N/A
Histone H2B	A0A125YW36	−1.100	N/A
Dense granule protein GRA1	A0A125YRV6	−1.037	N/A
Rhoptry protein ROP4	S7UI50	−1.024	N/A
Rhoptry protein ROP1	S7W7R3	−0.992	N/A
Putative peroxiredoxin 6	S7W128	−0.976	N/A
40S ribosomal protein S24	S7UQS1	−0.958	N/A
Histone H3	A0A125YSM0	−0.943	N/A
Succinate-CoA ligase, beta subunit	A0A125YHZ3	−0.917	N/A
Histone H4	A0A125YHU3	−0.857	N/A
Uncharacterized protein	S7W723	−0.818	N/A
Uncharacterized protein	A0A125YR22	−0.750	N/A
14-3-3 protein	A0A125YLJ3	−0.705	N/A
Ribosomal protein RPL19	A0A125YMW0	−0.651	N/A
Proliferation-associated protein 2G4	A0A125YVJ6	−0.504	N/A
Poly(ADP-ribose) glycohydrolase	S7W2K3	1.163	N/A
Uncharacterized protein	S7W7E3	1.207	N/A
IMP2_N domain-containing protein	S7WAV9	1.510	N/A
Acid phosphatase	S7V1S3	1.636	N/A

^1^ A modified *t*-test (two sided) with permutation-based FDR statistics (250 permutations, FDR = 0.05) was performed to compare the pyrimethamine-treated and vehicle (DMSO)-treated groups across three replicates. The fold changes (FCs) in the abundance of the nascent proteins (log_2_ scale) in the pyrimethamine-treated samples relative to the vehicle treatments across the three replicates are listed. N/A represents missing hits in the treatment groups.

## Data Availability

The mass spectrometry proteomics data have been deposited to the ProteomeXchange Consortium via the PRIDE partner repository with the dataset identifier PXD054994.

## Supplementary Materials

The following supporting information can be downloaded at: https://www.mdpi.com/article/10.3390/pathogens13100879/s1: Table S1: Newly synthesised *Toxoplasma gondii* proteins at 5, 10 and 50 µM pyrimethamine treatment for 1 h; Table S2: Protein-protein interaction network parameters of downregulated newly synthesised proteins upon pyrimethamine treatment (5 µM) in *Toxoplasma gondii*; Table S3: Protein-protein interaction network parameters of upregulated newly synthesised proteins upon pyrimethamine treatment (50 µM) in *Toxoplasma gondii*.

## References

[1] Montoya J.G., Liesenfeld O. (2004). Toxoplasmosis. Lancet.

[2] Heppler L.N., Attarha S., Persaud R., Brown J.I., Wang P., Petrova B., Tosic I., Burton F.B., Flamand Y., Walker S.R. (2022). The antimicrobial drug pyrimethamine inhibits STAT3 transcriptional activity by targeting the enzyme dihydrofolate reductase. J. Biol. Chem..

[3] Reynolds M.G., Oh J., Roos D.S. (2001). In vitro generation of novel pyrimethamine resistance mutations in the *Toxoplasma gondii* dihydrofolate reductase. Antimicrob. Agents Chemother..

[4] Montazeri M., Mehrzadi S., Sharif M., Sarvi S., Tanzifi A., Aghayan S.A., Daryani A. (2018). Drug Resistance in *Toxoplasma gondii*. Front. Microbiol..

[5] Garrison E.M., Arrizabalaga G. (2009). Disruption of a mitochondrial MutS DNA repair enzyme homologue confers drug resistance in the parasite *Toxoplasma gondii*. Mol. Microbiol..

[6] Shen B., Powell R.H., Behnke M.S. (2017). QTL Mapping and CRISPR/Cas9 Editing to Identify a Drug Resistance Gene in *Toxoplasma gondii*. J. Vis. Exp..

[7] Dieterich D.C., Link A.J., Graumann J., Tirrell D.A., Schuman E.M. (2006). Selective identification of newly synthesized proteins in mammalian cells using bioorthogonal noncanonical amino acid tagging (BONCAT). Proc. Natl. Acad. Sci. USA.

[8] Dieterich D.C., Lee J.J., Link A.J., Graumann J., Tirrell D.A., Schuman E.M. (2007). Labeling, detection and identification of newly synthesized proteomes with bioorthogonal non-canonical amino-acid tagging. Nat. Protoc..

[9] Ross P.L., Huang Y.N., Marchese J.N., Williamson B., Parker K., Hattan S., Khainovski N., Pillai S., Dey S., Daniels S. (2004). Multiplexed protein quantitation in Saccharomyces cerevisiae using amine-reactive isobaric tagging reagents. Mol. Cell Proteom..

[10] Wiese S., Reidegeld K.A., Meyer H.E., Warscheid B. (2007). Protein labeling by iTRAQ: A new tool for quantitative mass spectrometry in proteome research. Proteomics.

[11] Kalesh K., Denny P.W. (2019). A BONCAT-iTRAQ method enables temporally resolved quantitative profiling of newly synthesised proteins in Leishmania mexicana parasites during starvation. PLoS Negl. Trop. Dis..

[12] Kalesh K., Sundriyal S., Perera H., Cobb S.L., Denny P.W. (2021). Quantitative Proteomics Reveals that Hsp90 Inhibition Dynamically Regulates Global Protein Synthesis in Leishmania mexicana. mSystems.

[13] Koutsogiannis Z., Mina J.G., Albus C.A., Kol M.A., Holthuis J.C.M., Pohl E., Denny P.W. (2023). Toxoplasma ceramide synthases: Gene duplication, functional divergence, and roles in parasite fitness. FASEB J..

[14] de Lima Bessa G., Vitor R.W.A., Lobo L.M.S., Rego W.M.F., de Souza G.C.A., Lopes R.E.N., Costa J.G.L., Martins-Duarte E.S. (2023). In vitro and in vivo susceptibility to sulfadiazine and pyrimethamine of *Toxoplasma gondii* strains isolated from Brazilian free wild birds. Sci. Rep..

[15] Cox J., Mann M. (2008). MaxQuant enables high peptide identification rates, individualized p.p.b.-range mass accuracies and proteome-wide protein quantification. Nat. Biotechnol..

[16] Cox J., Neuhauser N., Michalski A., Scheltema R.A., Olsen J.V., Mann M. (2011). Andromeda: A peptide search engine integrated into the MaxQuant environment. J. Proteome Res..

[17] Tyanova S., Temu T., Sinitcyn P., Carlson A., Hein M.Y., Geiger T., Mann M., Cox J. (2016). The Perseus computational platform for comprehensive analysis of (prote)omics data. Nat. Methods.

[18] Szklarczyk D., Gable A.L., Lyon D., Junge A., Wyder S., Huerta-Cepas J., Simonovic M., Doncheva N.T., Morris J.H., Bork P. (2019). STRING v11: Protein-protein association networks with increased coverage, supporting functional discovery in genome-wide experimental datasets. Nucleic Acids Res..

[19] Shannon P., Markiel A., Ozier O., Baliga N.S., Wang J.T., Ramage D., Amin N., Schwikowski B., Ideker T. (2003). Cytoscape: A software environment for integrated models of biomolecular interaction networks. Genome Res..

[20] Holmes M.J., Shah P., Wek R.C., Sullivan W.J. (2019). Simultaneous Ribosome Profiling of Human Host Cells Infected with *Toxoplasma gondii*. mSphere.

[21] Wang X., Qu L., Chen J., Jin Y., Hu K., Zhou Z., Zhang J., An Y., Zheng J. (2023). Toxoplasma rhoptry proteins that affect encephalitis outcome. Cell Death Discov..

[22] Ibrahim H.M., Xuan X., Nishikawa Y. (2010). *Toxoplasma gondii* cyclophilin 18 regulates the proliferation and migration of murine macrophages and spleen cells. Clin. Vaccine Immunol..

[23] Narsimulu B., Qureshi R., Jakkula P., Singh P., Arifuddin M., Qureshi I.A. (2023). Exploration of seryl tRNA synthetase to identify potent inhibitors against leishmanial parasites. Int. J. Biol. Macromol..

[24] Houl J.H., Ye Z., Brosey C.A., Balapiti-Modarage L.P.F., Namjoshi S., Bacolla A., Laverty D., Walker B.L., Pourfarjam Y., Warden L.S. (2019). Selective small molecule PARG inhibitor causes replication fork stalling and cancer cell death. Nat. Commun..

[25] Kalesh K., Lukauskas S., Borg A.J., Snijders A.P., Ayyappan V., Leung A.K.L., Haskard D.O., DiMaggio P.A. (2019). An Integrated Chemical Proteomics Approach for Quantitative Profiling of Intracellular ADP-Ribosylation. Sci. Rep..

[26] Oliveira Souza R.O., Jacobs K.N., Back P.S., Bradley P.J., Arrizabalaga G. (2022). IMC10 and LMF1 mediate mitochondrial morphology through mitochondrion-pellicle contact sites in *Toxoplasma gondii*. J. Cell Sci..

[27] Samland A.K., Sprenger G.A. (2009). Transaldolase: From biochemistry to human disease. Int. J. Biochem. Cell Biol..

[28] Guiton P.S., Sagawa J.M., Fritz H.M., Boothroyd J.C. (2017). An in vitro model of intestinal infection reveals a developmentally regulated transcriptome of Toxoplasma sporozoites and a NF-kappaB-like signature in infected host cells. PLoS ONE.

[29] Wang S., Zhang Z., Wang Y., Gadahi J.A., Xu L., Yan R., Song X., Li X. (2017). *Toxoplasma gondii* Elongation Factor 1-Alpha (TgEF-1alpha) Is a Novel Vaccine Candidate Antigen against Toxoplasmosis. Front. Microbiol..

[30] Perez J.M., Siegal G., Kriek J., Hard K., Dijk J., Canters G.W., Moller W. (1999). The solution structure of the guanine nucleotide exchange domain of human elongation factor 1beta reveals a striking resemblance to that of EF-Ts from Escherichia coli. Structure.

[31] Plattner F., Yarovinsky F., Romero S., Didry D., Carlier M.F., Sher A., Soldati-Favre D. (2008). Toxoplasma profilin is essential for host cell invasion and TLR11-dependent induction of an interleukin-12 response. Cell Host Microbe.

[32] Tombacz K., Burgess G., Holder A., Werners A., Werling D. (2018). *Toxoplasma gondii* profilin does not stimulate an innate immune response through bovine or human TLR5. Innate Immun..

[33] Beck J.R., Chen A.L., Kim E.W., Bradley P.J. (2014). RON5 is critical for organization and function of the Toxoplasma moving junction complex. PLoS Pathog..

[34] Vaneynde P., Verbinnen I., Janssens V. (2022). The role of serine/threonine phosphatases in human development: Evidence from congenital disorders. Front. Cell Dev. Biol..

[35] Hortua Triana M.A., Marquez-Nogueras K.M., Vella S.A., Moreno S.N.J. (2018). Calcium signaling and the lytic cycle of the Apicomplexan parasite *Toxoplasma gondii*. Biochim. Biophys. Acta Mol. Cell Res..

[36] Rojas-Pirela M., Andrade-Alviarez D., Rojas V., Kemmerling U., Caceres A.J., Michels P.A., Concepcion J.L., Quinones W. (2020). Phosphoglycerate kinase: Structural aspects and functions, with special emphasis on the enzyme from Kinetoplastea. Open Biol..

[37] Li T., Guo Y. (2022). ADP-Ribosylation Factor Family of Small GTP-Binding Proteins: Their Membrane Recruitment, Activation, Crosstalk and Functions. Front. Cell Dev. Biol..

[38] Liendo A., Stedman T.T., Ngo H.M., Chaturvedi S., Hoppe H.C., Joiner K.A. (2001). *Toxoplasma gondii* ADP-ribosylation factor 1 mediates enhanced release of constitutively secreted dense granule proteins. J. Biol. Chem..

[39] Kremer K., Kamin D., Rittweger E., Wilkes J., Flammer H., Mahler S., Heng J., Tonkin C.J., Langsley G., Hell S.W. (2013). An overexpression screen of *Toxoplasma gondii* Rab-GTPases reveals distinct transport routes to the micronemes. PLoS Pathog..

[40] Das S., Hehnly H., Doxsey S. (2014). A new role for Rab GTPases during early mitotic stages. Small GTPases.

[41] Xue J., Jiang W., Li J., Xiong W., Tian Z., Zhang Q., Li S., Liu C., Huang K., Wang Q. (2019). *Toxoplasma gondii* RPL40 is a circulating antigen with immune protection effect. Folia Parasitol..

[42] Koonin E.V., Mushegian A.R., Tatusov R.L., Altschul S.F., Bryant S.H., Bork P., Valencia A. (1994). Eukaryotic translation elongation factor 1 gamma contains a glutathione transferase domain--study of a diverse, ancient protein superfamily using motif search and structural modeling. Protein Sci..

[43] Reis M., Alves C.N., Lameira J., Tunon I., Marti S., Moliner V. (2013). The catalytic mechanism of glyceraldehyde 3-phosphate dehydrogenase from Trypanosoma cruzi elucidated via the QM/MM approach. Phys. Chem. Chem. Phys..

[44] Kravets E., Degrandi D., Ma Q., Peulen T.O., Klumpers V., Felekyan S., Kuhnemuth R., Weidtkamp-Peters S., Seidel C.A., Pfeffer K. (2016). Guanylate binding proteins directly attack *Toxoplasma gondii* via supramolecular complexes. Elife.

[45] Nazarova L.A., Ochoa R.J., Jones K.A., Morrissette N.S., Prescher J.A. (2016). Extracellular *Toxoplasma gondii* tachyzoites metabolize and incorporate unnatural sugars into cellular proteins. Microbes Infect..

[46] Wang J.L., Li T.T., Zhang N.Z., Wang M., Sun L.X., Zhang Z.W., Fu B.Q., Elsheikha H.M., Zhu X.Q. (2024). The transcription factor AP2XI-2 is a key negative regulator of *Toxoplasma gondii* merogony. Nat. Commun..

